# Virtual Instruments for Peak-Overlapping Studies to Determine Low- and High-Concentration Components with Ion Chromatography: Potassium and Sodium

**DOI:** 10.3390/molecules29204882

**Published:** 2024-10-15

**Authors:** Nataša Gros

**Affiliations:** Faculty of Chemistry and Chemical Technology, University of Ljubljana, Večna Pot 113, SI-1000 Ljubljana, Slovenia; natasa.gros@fkkt.uni-lj.si; Tel.: +386-1-479-8555

**Keywords:** virtual instruments, peak overlapping, ion chromatography, sodium ion, potassium ion, seawater, Ringer’s solutions, citrate anticoagulant

## Abstract

We developed the LabVIEW-based virtual instruments (VIs) to bridge a gap in commercial software and to enable systematic peak-overlapping studies to recognise the concentration levels enabling reliable simultaneous determination of major and minor constituents in samples with wide concentration proportions. The VIs were applied to a case study of the ion chromatographic determination of potassium as minor and sodium as a major ion with an IonPac CS12A column and 50 μL injection loop. Two successive studies based on multilevel two-factorial response surface experimental designs, (1) a model peak-overlapping study based on single-ion injections, and (2) an accuracy and precision study, provided guidelines for real sample analyses. By adjusting sample dilutions so that the sodium mass concentration was set to 340 mg/L, the simultaneous determination of potassium in the presence of sodium was possible in samples with sodium over potassium concentration ratios between 14 and 341. The relative expanded uncertainty associated with potassium ion determination was between 0.52 and 4.4%, and the relative bias was between −3.8 and 1.9%. We analysed Ringer’s physiologic solutions, standard sea, trisodium citrate anticoagulant, and buffered citrate anticoagulant solutions. We confirmed that the VI-supported peak-overlapping studies contributed to the quality of results by enabling the evidence-based choices of concentration levels adjusted by a dilution.

## 1. Introduction

Liquid chromatography and other flow-based analytical methods require software for peak data evaluation. The common features are peak recognition, baseline settings, and peak area integration. The desired 1.5 resolution could not be achieved for all the peaks of interest under all circumstances. Post-acquisition signal processing can enhance peak separation and reduce peak width [1]. A quantitative analysis requires mathematical transformations that do not distort peak area and height, such as the derivative enhancement approach [2] and power-law-based [3,4] approaches.

The chromatographic resolution, *R*s = 1.5 assuming six standard-deviation retention time differences, corresponds to 0.1% overlap only for peaks of similar size and symmetrical Gaussian shape. Peak overlap prediction errors can be very substantial if the model does not hold [5]. Statistical moment analysis, not assuming idealised peak shapes, provides better insight.

Distorted peak shapes are observed in ion chromatography at high matrix concentrations [6,7,8]. Contrastingly to other chromatographic techniques, the matrix is not necessarily a generic name for the remaining sample composition potentially affecting the determination of the analyte. If the total ionic composition of a sample is an analytical objective, the minor and major constituents are both the analytes, but the latter can, at the same time, present a matrix effect to the former, depending on the elution sequence and the type of the major peak shape distortions.

Peaks of minor constituents might misleadingly seem unaffected if a chromatogram is observed on a full scale. Peak skimming is an imminent solution for riding peaks, a method choice affects the reliability of integration, and wrong decisions lead to an increase in error [9]. Deconvolution resolves the problem of partial peak overlapping as it is expressed but provides no general guidance.

The common ion concentrations can, in a range of real samples, vary several orders of magnitude. Samples with similar ion proportions present a similar case for ion chromatograph determination, they can be reduced to it by dilutions [10]. However, to the question of which concentrations should be set as a goal, there is no evident, simple, and single answer. With no systematic insight into the implications of different decisions, judgment is experientially based and not necessarily correct.

Peak-overlapping studies can provide an answer. Free area fraction as a single-peak-resolution criterion for mobile phase optimisation [11,12], even though derived from the peak overlapping consideration, is an entirely different approach with a different objective. The software that would bridge the gap is necessary.

A virtual instrument (VI) is a code that can operate modular hardware, acquire signals, read files, and modify, evaluate, present, and store data [13,14]. A VI can comprise all the functionalities or some of them, depending on the objectives. The VI is operated through a computer screen; its appearance with knobs, buttons, indicators, and numerical and graphical displays is similar to a real instrument but much easier to design, adjust, or re-structure.

The NI LabView is in contrast to text-based programming, a graphical programming language [15], and a VI development is drag-and-drop based. Pre-programmed functions and sub-routines, represented by icons, are available. A front panel, as a user interface, is designed first, and an executable code in the form of a block diagram is derived from it. Nodes comprising functions, sub-Vis, and structures are connected with wires that correspond to variables in a text-based programming language. The VIs reported in relation to liquid chromatography had different dedications, data acquisition [16,17], hardware control and data acquisition [18,19,20,21,22], detection [23,24], and numerical simulation [25].

In contrast to chromatography, zone overlapping, zone merging, and zone interpenetration model studies were used in other flow-based methods [26,27], but software to enable peak-overlapping evaluations was necessary. Kuljanin offered an initial solution by developing a VI to support zone penetration studies [28]. The disadvantages limiting its usability were as follows:

- No pre-treatment of raw data.
- No choice in the peak-recognition criteria.
- Not a general method for peak-overlapping evaluation, requires the user’s choice of the overlapping-pattern subcase.
- No aid for the output-data compilation to support more comprehensive studies.

Consequently, the independent, autonomous development of novel software was necessary to efficiently support systematic peak-overlapping studies. The reasons are that raw data pretreatment is usually necessary, a shift in a baseline level is not uncommon, correction of the peak data for a blank might be required, there might be artefacts or sections that are not relevant for the peak-overlapping study, and timelines might mismatch.

The research objectives of this paper can be expressed in four hypotheses (H1 to H4) and a research question Q1:

**H1.**  *The software, so-called virtual instruments (VIs), which we develop, can bridge the gap in the commercial chromatographic software and enable peak-overlapping studies.*

**H2.**  *The design of experiment 1—DoE 1-based VI-supported peak-overlapping studies enable the evidence-based recognition of the concentration levels and concentration proportions at which reliable simultaneous determination of the minor and the major ion can no longer be expected.*

**H3.**  *The design of experiment 2—DoE 2-based accuracy and precision studies respecting the evidence of the peak-overlapping study can test if the minor and major ion concentrations at the conditions foreseen as acceptable can be determined reasonably well.*

**H4.**  *The DoE 1- and DoE 2-derived conclusions enable reliable real sample analyses.*

Q1: What are the specific limiting conditions for the simultaneous determination of potassium as a minor and sodium as a major sample constituent for the specific IonPac CS12A column and experimental conditions that we used?

## 2. Results

**Figure 1:**
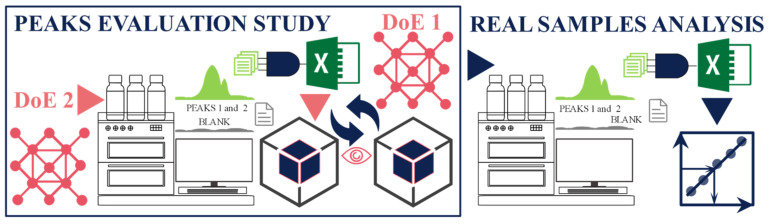
Three sets of ion chromatographic experiments, a design of experiment 1 (DoE 1)-supported peak-overlapping model study, a design of experiment 2 (DoE 2)-based peak accuracy and precision evaluation study, performed at peak overlapping of different extents; and real sample analysis exploiting the findings of the previous two studies.

The results were obtained from three sets of ion chromatographic experiments, as Figure 1 schematically indicates.

The first set of experiments is a model study of peak overlapping based on DoE 1 and employing the VIs. The second set of experiments, based on DoE 2, evaluates the impact of peak overlapping on the accuracy and precision of peak areas; the results of the real sample analyses follow.

A list of the essential abbreviations and their meanings is given in the Appendix A.

### 2.1. Results of Data Treatment with Virtual Instruments

Diverse terms are used in analytical techniques for a graphical representation of measured quantity recorded during time, e.g., chromatogram, SIA-gram, FIA-gram, etc. Since the VIs can treat any of them, we use a general term trace for any recording. We distinguish between a peak-trace and a blank-trace.

**Figure 2:**
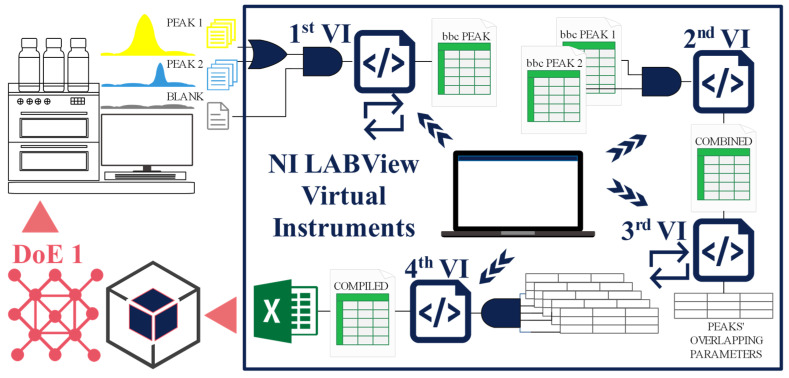
Peak-overlapping evaluation process, accomplished with four virtual instruments—VIs (enclosed). The first two are dedicated to data pretreatment; the third is the core, which carries out the overlapping evaluation; the fourth compiles the peak-overlapping-evaluation results of several experiments.

To evaluate peak overlapping, sodium peak traces, potassium peak traces, and a blank trace were recorded separately. Sodium elutes prior to potassium. Recordings stored in a text file format are raw data. Concentrations of sodium and potassium vary in a wide concentration range. The choice of concentrations was DoE 1 supported. The data treatment has been accomplished by four VIs operating in a sequence. A graphical overview is provided in Figure 2. The first two VIs perform data pre-treatment. The third VI outputs the final single-experiment peak-overlapping-related and peak-related results, and the fourth VI compiles the final output results of several related experiments to facilitate data analysis.

#### 2.1.1. The First Virtual Instrument—Raw Data Pretreatment

**Figure 3:**
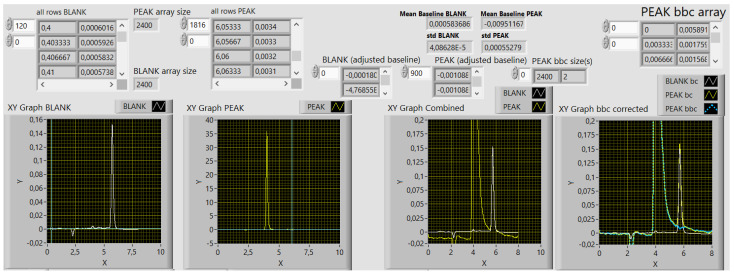
The front panel of the first virtual instrument with the results of raw data treatment. The first three XY graphs, from left to right, present raw input data, namely, a BLANK trace, a PEAK trace, and both in a combined view. The rightmost panel presents the baseline-corrected (bc) BLANK and PEAK traces and the baseline- and blank corrected (bbc) PEAK trace in light blue. The PEAK bbc array data (top right), related to the graph below, are exported as a text file. The values are raw, calculated, and the digits do not present the significant figures. Decimal sign is comma.

Figure 3 presents a section of the front panel of the 1st VI. The first two XY graphs, from left to right, present the raw data of a BLANK and a PEAK trace, respectively, as imported from the text files obtained with the PeakNet 5.21 chromatographic software. The traces can be inspected individually by changing the Y-axis scales. The next XY graph presents the traces in a comparison; consequently, nonzero and mismatched baselines become evident. The rightmost graph, in addition to the baseline-corrected traces (bc), also presents the baseline- and blank-corrected PEAK trace (bbc) in pale blue. It corresponds to the PEAK bbc array presented in the upper right corner. The data are transformed into strings and exported in a text format as a bbc PEAK output file, with the programmatically modified input name to support the data traceability.

#### 2.1.2. The Second Virtual Instrument—Peak Data Combined and Irregularities Repaired

**Figure 4:**
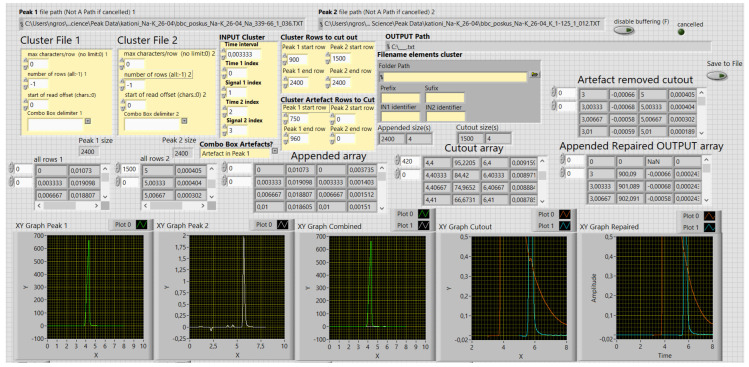
The front panel of the second virtual instrument. The first three XY graphs, from left to right, present the bbc-corrected-peak traces obtained in the previous stage. The next graph shows the cutout section, relevant to the peak-overlapping study. The rightmost graph, pertaining to the appended repaired OUTPUT array, had the artefact in the read trace removed. A common timeline was created by combining the times of both peaks’ traces. The missing response values were interpolated. The values are raw and calculated; the digits do not present the significant figures. Decimal sign is comma.

Figure 4 presents the front panel of the 2nd VI. The first three XY graphs, from left to right, present the imported data, in this case, bbc corrected PEAK 1 and PEAK 2 traces. The next graph presents the data cutouts relevant to the peak-overlapping study. In the range of the blue trace, there is an artefact on the declining side of the red trace; the rightmost graph shows that it was corrected for. This graph pertains to the appended repaired OUTPUT array above it, comprising data that are exported as a text file. The acquisition times of the traces were compared, if mismatched, combined, and missing peak response values interpolated. As a result, they both obtain a common timeline. The first column of the array comprises times and the next, relative times. The peak responses follow in the consecutive columns.

#### 2.1.3. The Third Virtual Instrument—Peak Parameters and Peak-Overlapping Parameters

**Figure 5:**
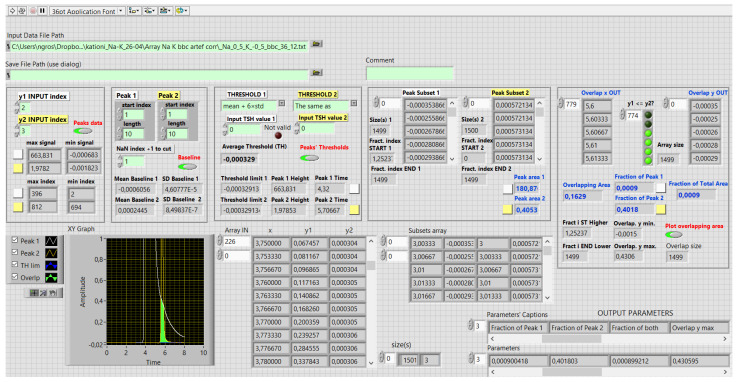
The front panel of the third virtual instrument with the results and graphical presentation of the peaks and peak-overlapping evaluation. The values are raw and calculated; the digits do not present the significant figures. Decimal sign is comma.

**Figure 6:**
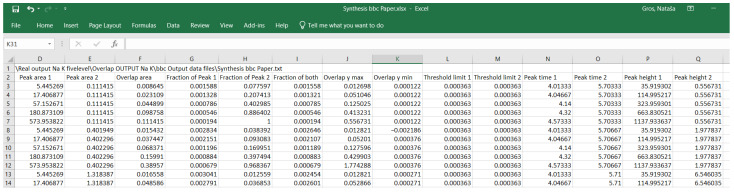
Output data of the third virtual instrument; each line corresponds to a separate experiment; the results pertaining to the same study were compiled with the fourth virtual instrument and exported as a text file, which was opened with MS Excel to be further analysed in relation to the design of experiment 1. The values are raw and calculated; the digits do not present the significant figures.

Figure 5 presents the front panel of the 3rd VI. The appended repaired two peak data with a common timeline are the input. In the XY graph is the final representation with the overlapping area marked in green. During the process, the graph changes, starting with the presentation of the input data. The indicators marked with white or yellow rectangles show the peak-evaluation or peak-overlapping-evaluation results. File export is optional, since one might test several different methods for threshold limit determination. We explain the output parameters in the following section, each line of the fourth VI’s MS Excel file in Figure 6 was extracted from the third VI’s output.

#### 2.1.4. The Fourth Virtual Instrument—Data Compilation

The table in Figure 6 is a compilation of the results of several experiments. There are fourteen output parameters; a data source file-path is not shown; the peak-related parameters are peak area, peak height, and peak time, corresponding to retention time in chromatography. The threshold limits of the peak recognition are given.

Peak-overlapping-related parameters are the overlap area corresponding to the area, presented in green, in Figure 6. The overlap area expressed relatively over the corresponding peak area, is denoted as a fraction of Peak 1 or 2, respectively, and defined by Equation (1). Index *i* indicates a peak number.

(1) $$Fraction\;of\;Peak\;i=\frac{Overlap\;area}{Peak\;area\;i}$$

Equation (2) defines the fraction of both as a fraction of the overlapping area over the total area under the curve outlining both peaks.

(2) $$Fraction\;of\;both=\frac{Overlap\;area}{\sum_{i=1}^{2}Peak\;area\;i-Overlap\;area}$$

Overlay y max and overlay y min are the maximal and the minimal responses of the overlap area, presented in green, in Figure 6.

### 2.2. Model Study of Peak Overlapping—Design of Experiments 1

**Figure 7:**
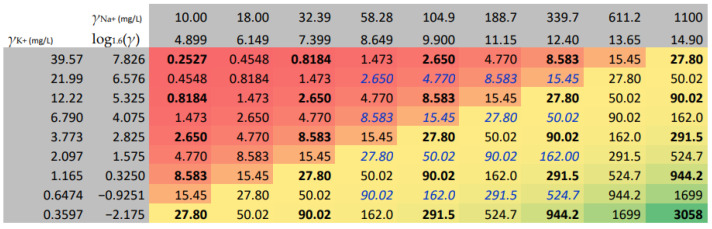
Mass concentrations of sodium and potassium and their corresponding 1.6-base logarithmic values as a foundation of the equidistant design of experiment 1 and design of experiment 2 factorial designs. The values in the coloured area are the sodium and potassium mass concentration ratios. The figures in bold correspond to the combinations of the factorial levels of the five-level design of experiment 1. The enframed, italicised figures in blue define a four-level design of experiment 2. The red–yellow–green colour gradient helps recognise similar values.

The DoE 1 is a two-factor, multilevel factorial design with 1 repetition, 2 blocks, and 50 runs. We confirmed that the 1.6-base logarithmic transformation of the ions’ mass concentrations enables the wide enough but equidistant sodium and potassium factor levels. Figure 7 presents the mass concentrations, their corresponding logarithmic values, and in the coloured area, the sodium to potassium mass concentration ratios. The red–yellow–green colour gradient helps recognise similar values. The whole lot of 18 single-ion solutions, injected into the chromatograph in duplicates, produced 36 files in total. The traces were treated with the 1st VI; two sets of 81 combinations of the sodium and potassium data were produced with the 2nd VI; and they formed the base for the DoE 1 to choose from. The selected combinations, marked in boldface, were treated with the third and fourth VI. From the listed parameters, the design’s output variables were derived.

Four output variables were evaluated with DoE 1, namely, Fraction Peak 2, Area1/Area2, Height1/Height2, and FractA/FractH. The first variable corresponds to the VI fraction of Peak 2. Index 2 pertains to potassium ion. The second and the third output variables are the quotients of Peak area 1 over Peak area 2 and, similarly, Peak height 1 over Peak height 2. The variable FractA/FractH is, by being a quotient of the previous two, an approximation of the relative change in the peak width of sodium ion over the peak width of potassium ion.

**Figure 8:**
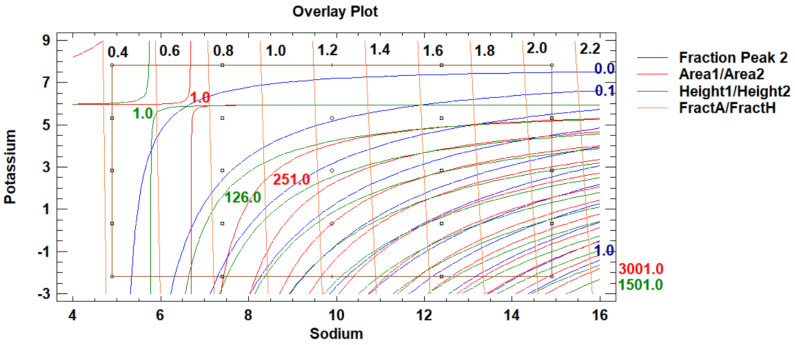
Overlay contour plot of response surfaces of the four output variables together with the experimental grid of the design of experiment 1. The sodium and potassium factor levels are expressed as a 1.6-base logarithm of mass concentrations.

Figure 8 presents the overlay contour plot of response surfaces of the four output variables, together with the experimental grid of the DoE 1. The scales of the experimental factors are logarithmic, as explained in relation to Figure 7. The quadratic models with 5 coefficients, as fitted, proved statistically significant at the 5.0% significance level. The R-squared values of the variables in the sequence, as previously named, were 88.85%, 64.35%, 74.38%, and 75.89%, respectively (more in Table S1).

In the bottom right corner of the DoE 1, we observe that Fraction peak 2 reaches 1, implying that the potassium peak area fully overlaps with the sodium peak area. A reliable determination of potassium concentration cannot be expected at the sodium over potassium concentration ratio of 3058 or close to it. Along the lowest potassium level, with an increase in sodium level, we notice the steepest ascent of Fraction peak 2, if compared with other potassium levels. As a result, Fraction peak 2 in the middle of the sodium range already reaches 0.5. The same is true if the highest sodium level is examined in the direction of the decreasing potassium level.

We also observe that with an increase in sodium level, the relative peak width ratio of sodium to potassium (FractA/FractH) changes from 0.4 to 2.1, indicating that sodium peak shape changes very severely, while we notice that the potassium-level decrease has a nearly negligible effect.

We can conclude that the highest sodium level and the lowest potassium level of DoE 1 are unfavourable for a reliable potassium determination. On the other hand, we can omit the highest potassium level since Fraction peak 2 is expected to be negligible at all sodium levels. The same is true for the lowest sodium level while at the second lowest sodium level the peak overlapping is still very mildly expressed.

As a result, we recognised the enframed, italicised figures in blue (Figure 7) as reasonable combinations of levels for the DoE 2.

### 2.3. Accuracy and Precision Study—Design of Experiments 2

The DoE 2 is a two-factor, multilevel factorial design with 16 runs with one sample to be taken during each run. The concentration ratios extend from 2.65 to 525. Eight single ion solutions, half comprising sodium and half potassium, with equal concentrations as specified for the combined solutions, were prepared as references. For all 24 solutions, chromatograms were recorded and peak areas, peak heights, and retention times were evaluated. The experiment was repeated for eight days. The mean values and standard deviations (*n* = 8) of all three parameters were calculated for the sodium and potassium peaks, respectively, pertaining to all the examined solutions. The results obtained for the combined solutions were evaluated against the results obtained for the single ion solutions of comparable concentrations, as the references.

Two relative parameters were derived, the relative accuracy (RA), abbreviated RAK or RANa, respectively, and the relative precision (RPc), abbreviated RPcK or RPcNa, each pair corresponding to potassium or sodium, ending in K or Na, respectively. It should be noted that accuracy is not considered as usual; it is a measure of how much the peak area, peak height, or retention time, obtained for an ion in the presence of another ion, deviates from the parameter obtained for a single ion of the same concentration in a solution with no potential interfering effect. The relative accuracy is a quotient of the mean value of the parameter, corresponding to a combined solution, over the mean value of the parameter, corresponding to a single ion solution. The relative precision is, similarly, the quotient of standard deviations.

The DoE 2 was applied to the relative accuracy of the sodium and potassium peak areas and to their relative precisions separately. In each case, the third variable Conc_ratio, which is the 1.6-base logarithmic value of the ratio of sodium over potassium mass concentration, was added as an aid in the interpretation of results, which we present as an overlay contour plot. Figure 7, through the logarithmic mass concentrations of the two ions, provides a link to the concentration rations.

The quadratic models with 4 coefficients have been fitted to the RA response variables. The *p*-values below 0.05 indicate that the models are statistically significant. The R-squared values of RAK_Area and RANa_Area were 71.40% and 52.32%, respectively (more in Table S1).

**Figure 9:**
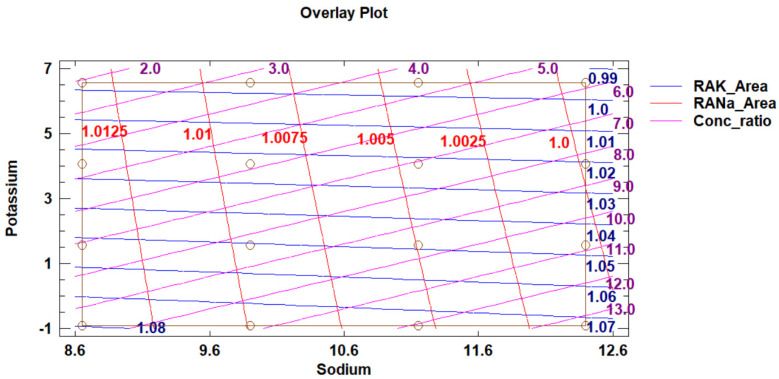
The overlay contour plot of response surfaces of the three output variables of the relative accuracy studies together with the experimental grid of the design of experiments 2, indicated with circles. The sodium and potassium factors-levels are expressed as 1.6-base logarithms of mass concentrations. The abbreviation RA stands for relative accuracy of either potassium (K) or sodium (*Na*) peak area (_Area). The variable Conc_ratio, which indicates the 1.6-base logarithmic value of the ratio of sodium over potassium mass concentration, is an aid to judge how a dilution of a particular sodium- and potassium-ion-containing solution can affect the results.

Figure 9 presents the overlay contour plot of response surfaces of the three output variables, together with the experimental grid of the DoE 2.

In Figure 9, we observe that at the highest potassium level, the RAK_Area value remains very close to one, confirming that there is a good agreement between the peak areas of potassium of the combined-ions solution or of the single-ion solution, no matter the sodium level. As the standardised Pareto chart also confirms, only the potassium factor level has a significant effect on the RAK_Area. The observation is in good agreement with what the DoE 1 indicates for the highest potassium level of the DoE 2, at which the peak overlapping is very weekly expressed. With a decrease in potassium level, the overestimation of the peak area of potassium in the presence of sodium increases. At the lowest potassium factor level, the RAK_Area even exceeds the value of 1.07.

For sodium, we similarly recognise that as the Pareto chart indicates, only the sodium ion factor level significantly affects the RANa_Area. At the lowest sodium level, the RANa_Area exceeds 1.0125, and with an increase in sodium level, the value decreases and, at the highest sodium level, reaches 1.00. The trend is similar to potassium but much weaker, and differences in RANa_Area values are small.

We can conclude that the factor levels of the DoE 2 were well-derived from the DoE 1 conclusions so that the reliable simultaneous determination of sodium and potassium mass concentrations can be considered possible in the whole range of samples with the sodium over potassium mass concentration ratios from 2.64 to 525. To enable the best conditions for sample-concentration-determinations, the most desirable are the levels as close to the right upper corner of DoE 2 as possible. If this is not feasible, the proximity to the highest potassium level should be set as a target.

The diagonal lines of the Conc_ratio are the guidelines for deciding how to set a sample dilution. They are the iso-sodium-over-potassium-concentration-ratio lines, expressed as the 1.6-base logarithms, and their corresponding antilogarithm values can be read from Table 1. By considering the sodium and potassium levels in the sample, in comparison with the levels of the DoE 2, one can, by appropriately choosing a dilution, influence where along the line the sample is going to be positioned and consequently affect the quality of the results.

The results of the relative precision study confirmed the standard deviations of the peak-areas were, for both ions, lower for the combined solutions if compared with the single-ion solutions.

### 2.4. Real Sample Analyses

To test the DoE-supported conclusions, we selected commercial and model samples with very different compositions, sodium and potassium ion concentrations, and concentration proportions so that the 1.6-base values of Conc_ratio varied between 5.6 and 12.4. The selected examples were, on one hand, sodium citrate anticoagulant solutions with the potassium in a very low concentration only potentially present as a contaminant, and on the other hand, standard sea [29] and Ringer’s isotonic physiological solutions [30] with potassium ions as the constituents.

To control the effect of the sodium peak shape on the determination of the potassium peak area we tended to reach very similar sodium concentrations in all the examined solutions by regulating the dilution. The target was set to 340.0 mg/L, which consequently also became the midpoint of the sodium calibration range. This concentration corresponds to the highest sodium factor level of the DoE 2. The decision follows the guidelines of the previous section.

The results were obtained within the two sets of experiments. The first, denoted by C2, contrastingly to C1, had higher concentrations of potassium calibration standards, covering the range 17.98 × (1 ± 0.667) mg/L. It comprises two calibration subsets to enable different checks and inter-comparisons. In the first subset, C2-S1, only the potassium concentration was increasing while the sodium concentration remained at 340.0 mg/L. In the second subset, C2-S2, concentrations of both ions were increasing simultaneously. The sodium calibration range was 340.0 × (1 ± 0.667) mg/L common to C2 and C1. In the latter, the potassium concentrations varied in the range of 1.978 (1 ± 0.818) mg/L, together with sodium. All the calibrations were five-point, with equidistant calibration levels. Each experiment was performed with single injections of calibration and examination solutions and repeated for five days. The final results of the five-day experiments were evaluated and compared.

A two-tailed F-test confirmed the heteroscedasticity of the C2-S2 calibration data of sodium (*p* = 3.2 × 10^−5^) and potassium (*p* = 0.013) at the 0.05 significance level but not of the C2-S1 calibration data of potassium (*p* = 0.094) and the C1 calibration data of sodium (*p* = 0.063) and potassium (*p* = 0.074). Consequently, the weighted least squares regression (WLS) or the ordinary least squares regression (OLS) were applied, as justifiable.

The parameters of the linear equations are summarised in Table 2. The symbols *a*, *b*, *s_y_*_/*x*_, and *R*^2^ denote intercept, slope, standard error of the estimate, and coefficient of determination, respectively. The MS Excel LINEST function was used for the OLS. For the WLS regression, a spreadsheet method of calculation was used. Equations from (3) to (9) were applied.

The *w_i_* in Equation (3) stands for weights, s_i_ is the standard deviation of the peak area, *y*_i_, ($i\in \;\left[1,n\right])$ was obtained during a five-day experiment for each of five (*n*) calibration levels (*x_i_*). The ${\bar{x}}_{w}$ and ${\bar{y}}_{w}$ in Equations (4) and (5) are the coordinates of the centroid. We understand the linear equation as *y* = *a* + *b* × *x*.

(3) $$w_{i}=\frac{s_{i}^{-2}\;n}{\sum_{i}^{n}s_{i}^{-2}}$$

(4) $${\bar{x}}_{w}=\frac{\sum_{i}^{n}w_{i}x_{i}}{n}$$

(5) $${\bar{y}}_{w}=\frac{\sum_{i}^{n}w_{i}y_{i}}{n}$$

Equations (6) and (7) indicate the calculation of the weighted slope and intercept.

(6) $$b_{w}=\frac{\sum_{i}^{n}{(w}_{i}x_{i}y_{i})-n{\bar{x}}_{w}{\bar{y}}_{w}}{\sum_{i}^{n}{(w}_{i}x_{i}^{2})-n{\bar{x}}_{w}^{2}}$$

(7) $$a_{w}={\bar{y}}_{w}-b_{w}{\bar{x}}_{w}$$

Equations (8) and (9) specify the calculation of the weighted standard error of the estimate and weighted correlation coefficient *r_w_* to calculate the weighted prediction coefficient R_w_^2^. The ${\hat{y}}_{i}$ are the fitted values corresponding to the calibration levels *x_i_*.

(8) $$s_{y/x\_w}=\sqrt{\frac{w_{i}{\left(y_{i}-{\hat{y}}_{i}\right)}^{2}}{n-2}}$$

(9) $$r_{w}=\frac{\sum_{i}^{n}w_{i}(x_{i}-{\bar{x}}_{w})(y_{i}-{\bar{y}}_{w})}{\sqrt{\sum_{i}^{n}w_{i}{\left(x_{i}-{\bar{x}}_{w}\right)}^{2}\cdot \sum_{i}^{n}w_{i}{\left(y_{i}-{\bar{y}}_{w}\right)}^{2}}}$$

To calculate the standard uncertainty (*s_x_*_0_) of the interpolated sodium or potassium ion concentration, *x*_0_, using the WLS linear equation, the weight (*w*_0_) has to be assigned to the corresponding peak area, *y*_0_. The model equations for potassium and sodium are given in Table 3, respectively. They were the best-fit models describing the dependence of the w_i_ on the *y*_i_.

Equations (10) and (11) enable the calculation of *s*_x0_ of the interpolated ion concentration for OLS or WLS regression, respectively. The symbol m corresponds to the number of repetitions of peak area measurements of the examined solution.

(10) $$s_{x0}=\frac{s_{y/x}}{b}\sqrt{\frac{1}{m}+\frac{1}{n}+\frac{{\left(y_{0}-\bar{y}\right)}^{2}}{b^{2}\sum_{i}^{n}{\left(x_{i}-\bar{x}\right)}^{2}}}$$

(11) $$s_{x0\_w}=\frac{s_{y/x\_w}}{b_{w}}\sqrt{\frac{1}{w_{0}}+\frac{1}{n}+\frac{{\left(y_{0}-{\bar{y}}_{w}\right)}^{2}}{{b_{w}}^{2}(\sum_{i=1}^{n}{w_{i}x_{i}}^{2}-n{\bar{x}}_{w}^{2})}}$$

Table 4 summarises the results of the C1 experiment. The symbol *U*_r_ designates the relative expanded standard uncertainty of interpolation, obtained from *s_x_*_0_ and *x*_0_ by applying the coverage factor *k* = 2. The symbols *V*_p_ and *V*_f_, by indicating the pipetted volume of the examined solution and the volume of a volumetric flask, define a dilution factor of each solution in particular. A mass concentration of an ion in an undiluted examined solution is marked by γ and its expected mass concentration by *τ*. The expression Δ/τ × 100 defines a relative bias; Δ indicates a difference between γ and τ. A quotient of the sodium and potassium ion mass concentrations, *γ*(Na^+^)/*γ*(K^+^), and its corresponding logarithmic value with the base 1.6, log_1.6_(Ratio), are given next. The last item in the table designated by log_1.6_(*x*_0_) indicates the logarithmic values of the interpolated mass concentrations of potassium and sodium ions.

The symbols BCT-C and BCT-B in Table 4 stand for the commercial citrate anticoagulant blood collection tubes (BCT), buffered and unbuffered, respectively. Abbreviations Na3Cit_V and CitBuff_V mark the verification solutions of trisodium citrate and buffered citrate solutions, respectively. All the successive verification solutions share a suffix_V. They are prepared from the NaCl and KCl standard substances only to reach the sodium and potassium concentrations matching the sample they are related to.

The results of the C2 experiment are presented in Table 5. The abbreviations STS, LR, AR, and RB stand for the standard sea, Ringer’s lactate solution, Ringer’s acetate solution, and Ringer Braun. The _V suffix denotes the verification standard solutions prepared from KCl and NaCl so that the potassium and sodium concentrations match the concentrations of the samples. The last two lines with the captions Ur Lower and |Bias|_r_ Lower indicate which calibration model for the determination of potassium, namely, the WLS or OLS, resulted in lower relative expanded uncertainty or lower absolute value of the relative bias.

## 3. Discussion

### 3.1. Real Sample Analyses

The C1 and C2 samples are due to their diversity in potassium-ion-concentration levels positioned distinctively differently along the rightmost edge of the DoE 2 (Figure 9). They form two separate groups towards the highest and the lowest potassium factor value, with a gap in between. This confirms that the samples were well selected.

Within the C2, the Standard Sea has the lowest position within the DoE 2 grid due to the highest *γ*(Na^+^)/*γ*(K^+^) ratio, close to 27, while the Ringer’s solution ratios range between approximately 22 and 14 (Table 5). The corresponding log_1.6_(Ratio) values by spanning from 7 to 5.6 define to which iso-Conc_ratio lines of the DoE 2 the samples belong (Figure 9). The C2 samples are within the area we recognised as the most desirable.

Differently, the C1 samples, by positioning themselves to the Conc_ratio lines with the values between 10.2 and 12.4, have a *γ*(Na^+^)/*γ*(K^+^) between 120 and 341 (Table 4). The latter ratio, pertaining to the Na3Cit_V, is the highest of all the samples and very close to the lowest bottom right corner of the DoE 2, assumed to be the least suited for a reliable potassium concentration determination.

By considering the samples in relation to the DoE 1 (Figure 8), we recognise that within the C2 group, the peak overlapping, expressed as Fraction Peak 2, is only between 0.05 and 0.15, in contrast to the C1 group’s values, which are much higher and between 0.4 and 0.55. Due to the fact that the calibration-solutions concentration levels stretch wider apart from those of the samples, Fraction Peak 2 ranges between 0.35 and 0.70 for the C1, contrastingly to the C2 for which the values extend between 0 and 0.2. Since Fraction Peak 2 increases with a decrease in potassium concentration within the C1 potassium ion calibration series quite severely, a higher uncertainty of the C1 calibration data can be assumed.

If we compare the relative expanded uncertainties (*U*_r_, *k* = 2) of interpolation of the potassium mass concentrations (*x*_0_) from the calibration line equation, we observe that the values of the C1 group are, by being between 1.6 and 4.4% (Table 4), distinctively higher than values of the C2 group, which did not exceed 1.0%. The 4.4% value corresponds to the Na3Cit_V, with the highest *γ*(Na^+^)/*γ*(K^+^) ratio of 341, the lowest determined potassium mass concentration (*x*_0_), and the highest extent of peak overlapping of 0.55, expressed as Fraction Peak 2. The *U*_r_ 4.4% of the C1 and the *U*_r_ 1.0% of the C2 both correspond to the samples, which were evaluated within the lower part of the calibration range at approximately 20% or 30% of the range, which is not much different from the *U*_r_ values.

However, there are dissimilarities within the C2 group regarding the confirmed regression model. The *U*_r_ values of the C2-S2 (WLS model), which range between 0.59 and 0.71%, are lower and less diversified than the values of the C2-S1 (OLS model), which extend between 0.52 and 1.0. The heteroscedastic nature of the data if not respected can cause positive systematic bias at low levels [31]; the neglect of weighting can increase the uncertainty of *x*_0_ at low levels for factor 10 [32].

There is also a difference in the determined concentrations of potassium. One-tail paired two-sample-for-mean *t*-test confirmed that the *x*_0_ values, which were obtained with the OLS regression, are significantly lower than those obtained with the WLR regression (*p* = 1.3 × 10^−6^).

The limits of detection (LoD) of potassium were 0.056, 0.13, and 0.17 mg/L, respectively, for C1, CS-S2, and CS-S1, as derived from the calibration parameters *s*_y/x_ and *b*. The corresponding values of the limits of quantification (LoQ) were 0.19, 0.43, and 0.55 mg/L.

### 3.2. Changes in Peak Shape, Retention Times, and Column Capacity

For a study of the reversed-phase behaviour influence on peak distortion, causing the minor ion retention time shift [8], we can realise that between 3.1 and 5.9% of the column capacity was exhausted, contrastingly to our case. With the highest 1100 mg/L sodium concentration, no more than 0.086% of the column capacity was occupied. The IonPac CS12A column stationary phase has relatively weak acidic carboxylic and phosphonic functionality, exhibiting high selectivity for hydronium ions. The ion exchange and reversed phase properties are observed.

By setting the criterion for Fraction of peak 2 not to exceed 0.7, the highest concentration of sodium for the DoE 2 was reduced to 340 mg/L. Moreover, the change in Area 1/Area 2 along the diagonal of the DoE 2 from the top left to the bottom right corner was still high, extending from 1 to 1700; the FractA/FractH as a measure of sodium over potassium peak width ratio was reduced to 1.7, which can be considered moderate if compared with the DoE 1 maximum 2.1, proving that the peak distortion was further limited. and consequently, the influence on the potassium retention times was very weakly expressed, confirming the reasonable conditions for quantitative determination.

### 3.3. Implications and Limitations

The VIs are generally applicable to peak-overlapping evaluations in flow-based methods and beyond.

The model peak-overlapping study approach that we suggested for ion chromatography contributes to evidence-based decisions in the simultaneous determination of minor and major constituents in wide concentration ratios and higher quality of results.

It should be noted that conclusions are not general but valid for a particular set of experimental conditions, a column choice, chromatographic software, and the applied integration algorithms. Comprehensive systematic peak-overlapping-based studies are rewarding if one frequently analyses several samples of different ion compositions with an equal experimental approach.

In our previous analysis of seawater and other saline solutions [33], we selected conditions that differ from what we would have selected on the basis of the current insight.

## 4. Materials and Methods

The section is organised into three subsections. In Section 4.1, we explain the essence of the operation of the VIs, which we developed, to enable the peak-overlapping studies.

The second section, Section 4.2, entitled Ion Exchange Chromatography, explains three sets of chromatographic experiments, schematically presented in Figure 1. The first experiment, Section 4.2.1, is a model study of peak overlapping. It is based on DoE 1 and employs the VIs to obtain the output data for the analysis. The second experiment, Section 4.2.2, based on DoE 2, evaluates the impact of peak overlapping on the accuracy and precision of peak area, height, and retention times of the two peaks. The third experiment, Section 4.2.3, is the application of the findings of the previous two experiments to real sample analyses.

The third section, Section 4.3, Designs of the experiment, defines DoE 1 and DoE 2.

### 4.1. Virtual Instruments for Peak-Overlapping Studies

The LabView 2015 software (Version 15.0f2, 32-bit), National Instruments Corporation (Austin, TX, USA) was used for programming. The process is accomplished with four VIs employed in a sequence. The third VI is the core, it evaluates the peak overlapping. The first two VIs ensure data pretreatment and the fourth VI posttreatment. The process is schematically presented in Figure 2.

The data acquired with an analytical instrument, the ion chromatograph in this case, and stored in text file format, are the raw data and a starting point. A file comprises two columns, acquisition times, expressed relatively, and their corresponding measurements. We distinguish a peak trace (PEAK) and a blank trace (BLANK). The latter is auxiliary if there is a need to correct a peak trace for it.

#### 4.1.1. The First Virtual Instrument—Raw Data Pretreatment

Within each peak-overlapping evaluation, the PEAK 1 and PEAK 2 raw-data text files are compulsory; the BLANK is optional. The procedure explained in the continuation is applied for each peak, respectively.

The VI reads the BLANK and PEAK data from text files; zeroes the baseline of both; corrects the PEAK data for a blank, if requested; and saves the corrected PEAK data into a corresponding bbc PEAK file. The abbreviation bbc stands for baseline and blank corrected. It could have been only bc—baseline corrected. The output file name is programmatically derived from a PEAK input file name to ensure data traceability.

#### 4.1.2. The Second Virtual Instrument—Peak Data Combined and Irregularities Repaired

The VI reads the bbc PEAK 1 and bbc PEAK 2 data out of the text files, obtained, respectively, in the previous stage. It creates an array comprising Time 1, Signal 1, Time 2, and Signal 2. The sections, which are relevant to the peak-overlapping study, are cut out of both data sets. The possible artefacts, if identified, can be corrected for.

During the data acquisition stage, the data are assumed to be acquired in regular pre-set intervals. Sometimes a personal computer as an acquisition device delays or omits some readings due to some other intermittent microprocessor tasks. Consequently, the dataset timelines mismatch. The counterparts of unmatched data are interpolated to correct for. The corrected, two-peak data sets with the common timeline are exported as the COMBINED output text file.

#### 4.1.3. The Third Virtual Instrument—Peak Parameters and Peak-Overlapping Parameters

The VI reads the data from the COMBINED text file and creates an array comprising Time, Signal 1, and Signal 2. Different methods can be selected to determine a peak threshold limit to recognise the start and end of each peak, respectively. The data sets between the earlier start and the later end are cut out, and the peak parameters and overlapping parameters are evaluated and stored in a text file format if requested. The output data comprise the input data file name and fourteen peak-overlapping- and peak-related parameters, which have already been explained in the Results section.

The enlarged, most essential front panel chromatograms of the first three virtual instruments are presented in Table S2.

#### 4.1.4. The Fourth Virtual Instrument—Data Compilation

The VI reads the peak-overlapping- and peak-related parameters out of the selected files and compiles the data to export as an output text file, called COMPILED, which can be read by MS Excel. By this process, the VI programmatically prepares a file with the data to be further analysed within the same study. In our case, it supports the DoE 1 data analysis.

#### 4.1.5. Experimental Data Treated with the Virtual Instruments

Eighteen single-ion solutions, half containing potassium ions and half sodium ions, with mass concentrations as specified in Figure 7, were injected into an ion chromatograph. The conditions were the same as described in the next section. The experiment, which was carried out twice, resulted in 36 chromatograms, which were treated with the first VI. The 81 different combinations of sodium and potassium concentration levels are possible, requiring 162 data treatments with the second VI. Out of them, 25 × 2 output data files were selected as the DoE 1 requested and treated with the third and fourth VI.

### 4.2. Ion Exchange Chromatography

The DX-500 Ion Chromatograph (Dionex Corporation, Sunnyvale, CA, USA) consisted of the GP-40 Gradient Pump and the ED-40 Electrochemical detector with a conductometric cell. A 50-µL injection loop, IonPac CS12A (4 × 250 mm) column, IonPac CG12A (4 × 250 mm) guard column, and CSRS 300 (4 mm) suppressor in closed mode of operation were used (Thermo Fisher Scientific, Waltham, MA, USA). The current was set to 50 mA. The eluent was 22 mmol/L methanesulfonic acid, CH_3_SO_3_H (*M* = 96.11 g/mol, *w* ≥ 0.990, *ρ* = 1.481 g/cm^3^ at 25 °C, CAS: 75-75-2, Sigma Aldrich, Steinheim, Germany), flow rate was 1 mL/min. The PeakNet 5.21 chromatographic software was used. At 5 min, the Tangent Skim Riders on Tailing Slope and Halve Bunching Factor were applied if the preceding sodium peak affected the potassium peak recognition. The time function peak width (PW) for potassium was adjusted as the chromatogram required.

Standard substances, potassium chloride, KCl ((*M* = 74.55 g/mol, 0.990 ≤ *w* ≤ 1.005), Fluka, Seelze, Germany) and sodium chloride NaCl ((*M* = 58.44 g/mol, *w* ≥ 0.995), Merck, Darmstadt, Germany) were dried at 110 °C for two hours.

Deionised water, additionally purified through the Milli-Q system (Millipore, Billerica, MA, USA), was used to prepare all solutions.

#### 4.2.1. A Model Study of Peak-Overlapping

A study required several related experiments. Within a single experiment, the overlapping of the peaks of two different species is examined. Concentrations of the species are specific; other experimental conditions are general. The data acquired for a blank and each of the species, namely, sodium and potassium, separately in a time domain, and saved in text-file format, respectively, are the initial VI inputs.

Five potassium ion calibration solutions, prepared from KCl, had concentrations of 0.3597, 1.165, 3.773, 12.22, and 39.57 mg/L. Concentrations of five sodium ion calibration solutions prepared from NaCl were 10.00, 32.39, 104.9, 339.7, and 1100 mg/L, respectively. Each solution was injected into an ion chromatograph in a duplicate. The injection sequence was potassium solution prior to sodium solution, and both were in ascending concentration order.

#### 4.2.2. Accuracy and Precision Study

A stock standard solution with a potassium ion concentration of 1.2 g/L was prepared from KCl. A stock standard solution with a sodium ion concentration of 10 g/L was prepared from NaCl. Among the derived working standard solutions, 16 contained potassium and sodium, 4 contained potassium only, and 4 sodium only. The concentration levels of potassium were 0.647, 2.097, 6.790, and 21.99 mg/L. The sodium concentration levels were 58.28, 104.9, 188.7, and 339.7 mg/L. The experiment was repeated for eight days, with single injections of all 24 working solutions. The sequence was potassium-only solutions first in ascending order of their ion concentration. The remaining standards were arranged by the sodium level as the first criterion and potassium as the second, both in increasing order. Consequently, within each subgroup of solutions with equal sodium ion concentration, the first in a sequence was always the solution with no added potassium stock standard.

#### 4.2.3. Real Sample Analyses

The commercial samples were:

- Ringer Braun lactate solution, abbreviated RB (Ringerjeva raztopina Braun, raztopina za infundiranje, 500 mL, p.n. 363 2431, Lot 232158142, B.Braun Melsungen AG, Melsungen, Germany);
- Citrate anticoagulant blood collection tubes (109 mmol/L, 1.8 mL), abbreviated BCT-B (Vacutube, LT Burnik d.o.o., Skaručna, Slovenia);
- Buffered citrate anticoagulant blood collection tubes (109 mmol/L, 1.8 mL), abbreviated BCT-C (BD Vacutainer^®^, Becton, Dickinson and Company (BD), Franklin Lakes, NJ, USA).

A list of the additional chemicals used in the preparation of model samples and verification solutions comprises the following:

- Magnesium chloride hexahydrate, MgCl_2_·6H_2_O ((*M* = 203.30 g/mol, *w* ≥ 0.99, pro analysi), Gram-Mol, Zagreb, Croatia);
- Calcium chloride dihydrate, CaCl_2_·2H_2_O ((*M* = 147.02 g/mol, *w* ≥ 0.995, GR), Merck, Darmstadt, Germany);
- Sodium sulfate, Na_2_SO_4_ ((*M* = 142,04 g/mol, *w* ≥ 0.99, pro analysi), Merck, Darmstadt, Germany);
- Sodium hydrogen carbonate, NaHCO_3_ ((*M* = 84.01 g/mol, *w* ≥ 0.997), Riedel-de Haën, Seelze, Germany);
- Sodium acetate trihydrate, CH_3_COONa·3H_2_O ((*M* = 136.08 g/mol, 1.005 ≥ *w* ≥ 0.995, pro analysi), E. Merck, Darmstadt, Germany);
- DL-lactic acid Na-salt, NaC_3_H_5_O_3_ ((*M* = 112.1 g/mol, cryst. research grade), Serva, Feinbiochemica, Heidelberg, Germany/New York, NY, USA).

The masses of chemicals weighed for 100 mL of model-sample solutions and verification solutions are summarised in Table 6. The symbols are explained in Section 2.4.

We founded the preparation of the standard sea model-sample solution (STS) on the literature data [29]. We based the preparation of the Ringer’s lactate (LR) and Ringer’s acetate (LA) model-sample solutions on the composition of commercial products.

Verification solutions, denoted with the _V suffix, were prepared from the KCl and NaCl standards and dried for two hours at 110 °C. They match the sodium and potassium ion concentrations of the samples. In setting the sodium level of the sodium citrate buffer for blood collection tubes (CitBuff_V), we relied on the US Patent US5667963A. The sodium ion content of the trisodium citrate verification solution (Na3Cit_V) was derived from the 109 mmol/L citrate anticoagulant solution. In setting the levels of potassium ions, we relied on previous experiences.

### 4.3. Designs of Experiments

The designs are two-factorial. The factors are the sodium and potassium mass concentrations, expressed as the 1.6-base logarithm to enable wide enough concentration ranges while keeping equidistant factorial levels. More details are given in Figure 7. The STATGRAPHICS Centurion XVI, software (Version 16.2.04 (64-bit), StatPoint Technologies, Inc., Warrenton, VA, USA) was used.

#### 4.3.1. Design of Experiment 1

The DoE 1 is a two-factorial, five-level response-surface design with 1 repetition, 2 blocks, and 50 runs. Quadratic statistical models have been fit to the four response variables (Figure 8). The source data are the VI-treated (Section 4.1) and chromatographic data (Section 4.2.1).

#### 4.3.2. Design of Experiment 2

The DoE 2 is a two-factor four-level response-surface design with 16 runs. Quadratic statistical models have been fit to two sets of three response variables, respectively (Figure 9). The source data are the chromatographic data obtained as described in Section 4.2.2.

## 5. Conclusions

**H1.**  *We confirmed that the software, so-called virtual instruments (VIs), which we develop, can bridge the gap in the commercial chromatographic software and enable peak-overlapping studies or support zone penetration studies in other flow-based methods.*

**H2.**  *The Design of Experiment 1—DoE 1-based VI-supported peak-overlapping studies enable the evidence-based recognition of the concentration levels and concentration proportions at which reliable simultaneous determination of the minor and the major ion can no longer be expected. We recognised the sodium ion concentration of 340 mg/L as a limit above which the major-peak shape distortions become too intensive. The judgement was founded on the requirements, first, that the major over minor peak width ratio does not exceed 1.7 and second, that the overlap-fraction of the minor peak area does not exceed 0.7.*

**H3.**  *The Design of Experiment 2—DoE 2-based accuracy and precision study based on the conclusions of the peak-overlapping study suggested at which concentration levels the minor and major ion concentrations can be determined successfully. The range extends between the major over the minor constituent mass concentration ratio 2.65 and 525, at mass concentrations of potassium and sodium ion changing from 0.65 to 22.0 mg/L and from 58.3 to 340 mg/L, respectively.*

**H4.**  *On the examples of citrate anticoagulant solutions, standard seawater, and Ringer’s solutions, we confirmed that the DoE 1- and DoE-2 derived conclusions enabled reliable real sample analyses.*

Q1: For the IonPac CS12A column and experimental conditions, which we used, we confirmed that the simultaneous determination of potassium as a minor and sodium as a major constituent was possible in samples with the sodium over potassium concentration proportions between 13.9 and 341. Concentration levels of sodium and potassium, shifted towards the right side of the DoE 2 overlay plot, preferably as close as possible to the right upper corner, with the sodium mass concentration close to the 340 mg/L limit, are favourable. By adjusting the dilution, we regulated the sodium concentration level in the analysed samples so that it was as close to the target as possible. By adjusting the concentration level of a major constituent, we can analyse several samples with different major over minor ion mass concentration ratios under similar conditions, and we gain control over the effect of the major peak shape distortion on the integration of the minor peak area. The approach in which we only determined potassium concertation and kept the sodium level in all the potassium calibration standards constant, at the value of 340 mg/L, did not prove superior to the approach in which we determined the sodium and potassium ion concentrations simultaneously.

## Figures and Tables

**Table 1:** Transformation of the 1.6-base logarithmic values of the ratio of sodium over potassium mass concentration (Conc_ratio) to the corresponding antilogarithm values.

2	3	4	5	6	7	8	9	10	11	13	13
2.56	4.10	6.55	10.5	16.8	26.8	42.9	68.7	110	176	281	450

**Table 2:** Calibration parameters of the *y* = *a* + *b* × *x* calibration model (*n* = 5) of the C1 and C2 experiments, fitting the potassium and sodium calibration data by the ordinary least squares regression (OLS) or the weighted least squares regression (WLS), as more justifiable.

Parameters	*a*	*b*	*s_y/x_*	*R* ^2^	*a*	*b*	*s_y/x_*	*R* ^2^
Analyte	Potassium				Sodium			
C1 (OLS)	16 × 10^3^	236 × 10^3^	4402	0.9998	1.1 × 10^6^	363 × 10^3^	408.8 × 10^3^	1.0000
C2-S2 (WLS)	5 × 10^3^	232.1 × 10^3^	9874	0.9997	0.97 × 10^6^	360 × 10^3^	81.94 × 10^3^	1.0000
C2-S1 (OLS)	5 × 10^3^	234.6 × 10^3^	12.95 × 10^3^	1.0000	/	/	/	/

**Table 3:** Equations to assign the *w*_0_ to the peak area, *y*_0_, corresponding to the ion in the examined solution.

Analyte	Potassium	Sodium
C2-S2 (WLS)	*w*_0_ = −1.66 × ln(*y*_0_) + 26.1 *R*^2^ = 0.8953	*w*_0_ = 15.3 × e*^k^*^×*y*0^; *k* = −3.35 × 10^−8^ *R*^2^ = 0.9444

**Table 4:** Sodium and potassium ion concentrations obtained in the C1 experiment with the ordinary least squares regression.

Solution	Na3Cit_V	CitBuff_V	BCT-C	BCT-B	Na3Cit_V	CitBuff_V	BCT-C	BCT-B
Analyte	Potassium				Sodium			
*x*_0_ (mg/L)	0.993	2.703	2.148	<0.360	338.8	339.0	258.8	235.4
*s*_x0_ (mg/L)	0.021	0.021	0.020	/	1.2	1.2	1.3	1.3
*U*_r_, *k* = 2 (%)	4.4	1.6	1.9	/	0.73	0.73	0.97	1.1
*V*_p_ (mL) **	2.25	2.65	10 *	10 *	2.25	2.65	10 *	10 *
*V*_f_ (mL) ***	50	50	50	50	50	50	50	50
*γ* (mg/L)	22.1	51.0	5.37	/	7.53 × 10^3^	6.40 × 10^3^	647	588
*τ* (mg/L)	21.97	51.96	/	/	7499	6335	/	/
Δ/*τ* × 100 (%)	0.41	−1.8	/	/	0.39	0.96	/	/
*γ*(Na^+^)/*γ*(K^+^)	341	125	120	/	341	125	120	/
log_1.6_(Ratio)	12.41	10.28	10.19	/	12.41	10.28	10.19	/
log_1.6_(*x*_0_)	−0.02	2.12	1.63	/	12.394	12.396	11.82	11.62

* Composed sample prepared from 10 blood collection tubes. ** Volumes are given as nominal; they were measured with the Transferpette S (500–5000 μL) pipette, BRAND^®^. *** Volumes are nominal; volumetric flasks were A-class.

**Table 5:** Results of the C2 experiment for the sodium and potassium concentration determination in samples and verification solutions obtained with the ordinary least squares regression (OLS) or the weighted least squares regression (WLS).

**Solution**	**STS_V**	**LR_V**	**AR_V**	**RB_V**	**STS**	**LR**	**AR**	**RB**
Analyte	Potassium—calibration potassium and sodium (C2-S2), data heteroscedastic, WLS							
*x*_0_ (mg/L)	12.726	24.361	18.531	15.251	12.810	23.576	17.722	16.112
*s*_x0_ (mg/L)	0.041	0.086	0.055	0.046	0.042	0.079	0.052	0.048
*U*_r_, *k* = 2 (%)	0.65	0.71	0.59	0.61	0.65	0.67	0.59	0.60
*V*_p_ (mL) *	3.15	2.25	2.25	5	3.15	2.25	2.25	5
*V*_f_ (mL) **	100	20	20	50	100	20	20	50
*γ* (mg/L)	404.0	216.5	164.7	152.5	406.7	209.6	157.5	161.1
*τ* (mg/L)	402.3	212.9	161.6	156.4	406.6	209.3	156.4	/
Δ/*τ* × 100 (%)	0.42	1.7	1.9	−2.5	0.023	0.15	0.71	/
*γ*(Na^+^)/*γ*(K^+^)	26.84	13.91	18.27	22.27	27.1	14.23	18.87	21.46
log_1.6_(Ratio)	7.001	5.601	6.182	6.603	7.017	5.650	6.250	6.524
log_1.6_(*x*_0_)	5.414	6.793	6.211	5.797	5.426	6.724	6.116	5.914
**Solution**	**STS_V**	**LR_V**	**AR_V**	**RB_V**	**STS**	**LR**	**AR**	**RB**
Analyte	Sodium—calibration potassium and sodium (C2-S2), data heteroscedastic, WLS							
*x*_0_ (mg/L)	341.56	338.77	338.65	339.69	346.56	335.56	334.43	345.73
*s*_x0_ (mg/L)	0.49	0.48	0.48	0.48	0.50	0.47	0.47	0.50
*U*_r_, *k* = 2 (%)	0.29	0.28	0.28	0.28	0.29	0.28	0.28	0.29
*V*_p_ (mL) *	3.15	2.25	2.25	5	3.15	2.25	2.25	5
*V*_f_ (mL) **	100	20	20	50	100	20	20	50
*γ* (mg/L)	10.84 × 10^3^	3011	3010	3397	11.00 × 10^3^	2983	2973	3457
*τ* (mg/L)	10.76 × 10^3^	2988	2987	3375	10.97 × 10^3^	2989	2989	/
Δ/*τ* × 100 (%)	0.80	0.78	0.77	0.64	0.27	−0.23	−0.54	/
*γ*(Na^+^)/*γ*(K^+^)	26.84	13.91	18.27	22.27	27.1	14.23	18.87	21.46
log_1.6_(Ratio)	7.001	5.601	6.182	6.603	7.017	5.650	6.250	6.524
log_1.6_(*x*_0_)	12.412	12.394	12.393	12.400	12.443	12.374	12.367	12.437
**Solution**	**STS_V**	**LR_V**	**AR_V**	**RB_V**	**STS**	**LR**	**AR**	**RB**
Analyte	Potassium—calibration potassium, sodium constant (C2-S1), data homoscedastic, OLS							
*x*_0_ (mg/L)	12.552	24.076	18.298	15.052	12.636	23.290	17.497	15.903
*s*_x0_ (mg/L)	0.063	0.063	0.060	0.061	0.062	0.062	0.060	0.061
*U*_r_, *k* = 2 (%)	1.0	0.52	0.66	0.81	1.0	0.54	0.69	0.76
*V*_p_ (mL) *	3.15	2.25	2.25	5	3.15	2.25	2.25	5
*V*_f_ (mL) **	100	20	20	50	100	20	20	50
*γ* (mg/L)	398.5	213.9	162.6	150.5	401	207.0	155.5	159.0
*τ* (mg/L)	402.3	212.9	161.6	156.4	406.6	209.3	156.4	/
Δ/*τ* × 100 (%)	−0.94	0.47	0.62	−3.8	−1.3	−1.1	−0.57	/
*γ*(Na^+^)/*γ*(K^+^)	27.2	14.08	18.51	22.6	27.4	14.41	19.11	21.74
log_1.6_(Ratio)	7.03	5.626	6.208	6.631	7.05	5.676	6.277	6.552
log_1.6_(*x*_0_)	5.38	6.767	6.185	5.768	5.40	6.698	6.090	5.886
*U*_r_ Lower	WLS	OLS	WLS	WLS	WLS	OLS	WLS	WLS
*|Bias|*_r_ Lower	WLS	OLS	OLS	WLS	WLS	WLS	OLS	/

* Volumes are given as nominal; they were measured with the Transferpette S (500–5000 μL) pipette, BRAND^®^. ** Volumes are nominal; volumetric flasks were A-class.

**Table 6:** Masses of chemicals in g, weighed to prepare 100 mL of model-sample solutions and verification solutions (_V suffix).

Solution	STS	STS_V	LR	LR_V	AR	AR_V	RB_V	CitBuff_V	Na3Cit_V
KCl	0.0759	0.0769	0.0400	0.0407	0.0299	0.0309	0.0299	0.0099318 *	0.0041990 *
NaCl	2.3919	2.7415	0.6001	0.7615	0.5860	0.7613	0.8602	1.6145	1.9112
MgCl_2_·6H_2_O	1.0734	/	/	/	0.0209	/	/	**/**	**/**
CaCl_2_·2H_2_O	0.1512	/	0.0273	/	0.0289	/	/	**/**	**/**
Na_2_SO_4_	0.4009	/	/	/	/	/	/	**/**	**/**
NaHCO_3_	0.0143	/	/	/	/	/	/	**/**	**/**
CH_3_COONa·3H_2_O	/	/	/	/	0.4080	/	/	**/**	**/**
NaC_3_H_5_O_3_	/	/	0.3094	/	/	/	/		
*V*_solution_ (mL)	100.0038 **	100	100	100	100	100	100	100	100

* Weighing with a balance with the readability of 0.1 μg, instead of 0.1 mg, as valid for all other cases. ** Solution mass in g, instead of volume (density of seawater at 23 °C is 1.024 kg/L).

## Data Availability

The original contributions presented in the study are included in the article/Supplementary Materials, further inquiries can be directed to the corresponding author.

## Supplementary Materials

The following supporting information can be downloaded at: https://www.mdpi.com/article/10.3390/molecules29204882/s1, Table S1: Designs of experiments; Table S2: Enlarged essential chromatograms of the virtual instrument front panels.

## Appendix A

The essential abbreviations are explained in Table A1.

**Table A1:** A list of the essential abbreviations and their meaning.

Abbreviation	Meaning
*a*	Intercept.
AR	Ringer’s acetate solution.
AR_V	Verification Ringer’s acetate solution.
Area1/Area2	Quotient of Peak area 1 over Peak area 2.
*b*	Slope.
BCT	Citrate anticoagulant blood collection tube.
BCT-B	Citrate anticoagulant blood collection tube—unbuffered.
BCT-C	Citrate anticoagulant blood collection tube—buffered.
*|Bias|*_r_ Lower	Lower absolute value of the relative bias.
CitBuff_V	Verification buffered citrate solution.
Conc_ratio	1.6-base logarithmic value of the ratio of sodium over potassium mass concentration.
DoE	Design of experiment.
FractA/FractH	Relative change in the peak width of sodium ion over the peak width in potassium ion.
Fraction Peak 2	Overlap area expressed relatively over the Peak area 2.
Height1/Height2	Quotient of Peak height 1 over Peak height 2.
*k* = 2	Coverage factor.
log_1.6_(Ratio)	Logarithmic values of the quotient of the sodium and potassium ion mass concentrations.
log_1.6_(*x*_0_)	Logarithmic values of the interpolated mass concentrations of potassium and sodium ions.
LR	Ringer’s lactate solution.
LR_V	Verification Ringer’s lactate solution.
Na3Cit_V	Verification trisodium citrate solution.
OLS	Ordinary least squares regression
RAK_Area	Relative accuracy of potassium peak area.
RANa_Area	Relative accuracy of sodium peak area.
RB	Ringer Braun solution.
RB_V	Verification Ringer Braun solution.
STS	Standard sea.
STS_V	Standard sea verification solution.
*s*_x0_	Standard uncertainty (*s*x_0_) of the interpolated sodium or potassium ion concentration, x_0_.
*U*_r_	Relative expanded standard uncertainty of interpolation.
*U*_r_ Lower	Lower relative expanded uncertainty.
*V*_f_	Volume of volumetric flask.
VI	Virtual instrument.
*V*_p_	Pipetted volumes.
WLS	Weighted least squares regression.
*x*_0_	Interpolated sodium or potassium ion mass concentration.
*γ*	Mass concentration of ions in examined solution.
*γ*(Na^+^)/*γ*(K^+^)	Quotient of the sodium and potassium ion mass concentrations.
Δ/*τ* × 100	Relative bias.
*τ*	Expected mass concentration.
