# Supplementary material for: Virtual Instruments for Peak-Overlapping Studies to Determine Low- and High-Concentration Components with Ion Chromatography: Potassium and Sodium
Source: Molecules. 2024 Oct 15;29(20):4882. doi: 10.3390/molecules29204882 (PMC11510477; doi:10.3390/molecules29204882)

Table S1: Designs of experiments

Design of Experiments 1

Model	*Fraction Peak 2*	*Area1/Area2*	*Height1/Height2*	*FractA/FractH*
Transformation	none	none	none	none
Model d.f.	3	3	3	3
P-value	0.0000	0.0000	0.0000	0.0000
Error d.f.	45	45	45	45
Stnd. error	0.105356	657.355	243.04	0.346926
R-squared	88.85	64.35	74.38	75.89
Adj. R-squared	87.86	61.18	72.10	73.74

				Power at	*Power at*	*Power at*
Coefficient	*Standard Error*	*VIF*	*Ri-Squared*	*SN = 0.5*	*SN = 1.0*	*SN = 2.0*
A	0.2	1.0	0.0	23.14%	68.67%	99.83%
B	0.2	1.0	0.0	23.14%	68.67%	99.83%
AB	0.282842	1.0	0.0	13.92%	40.92%	93.31%

alpha = 5.0%, sigma estimated from total error with 45 d.f.

Standardized Pareto Chart for Fraction Peak 2

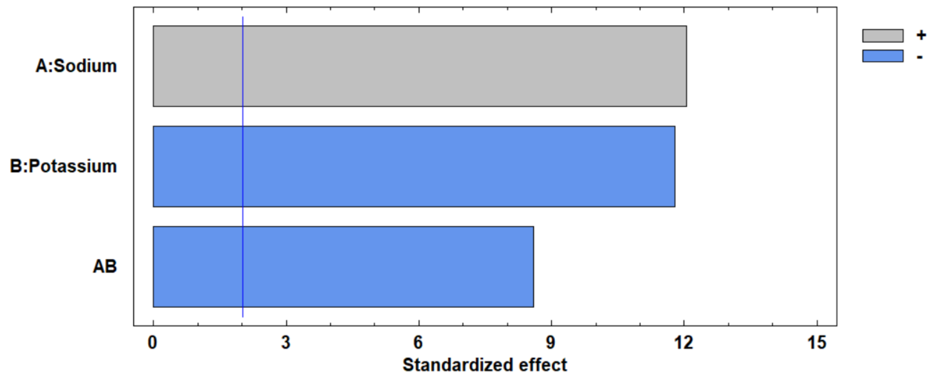

Standardized Pareto Chart for Area1/Area2

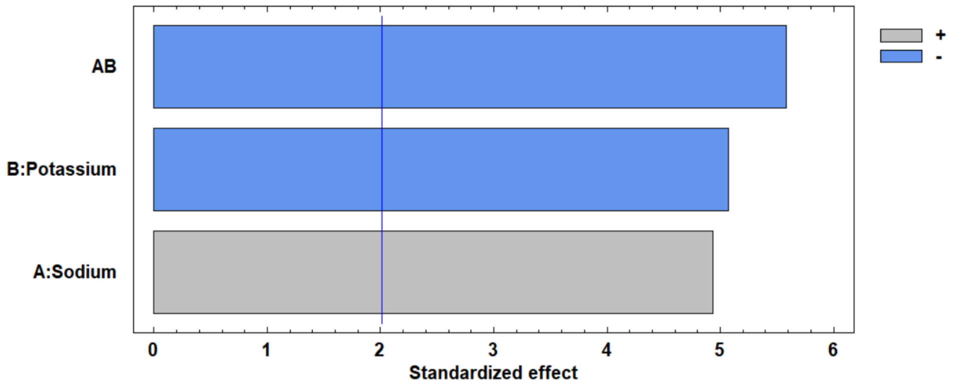

Standardized Pareto Chart for Height1/Height2

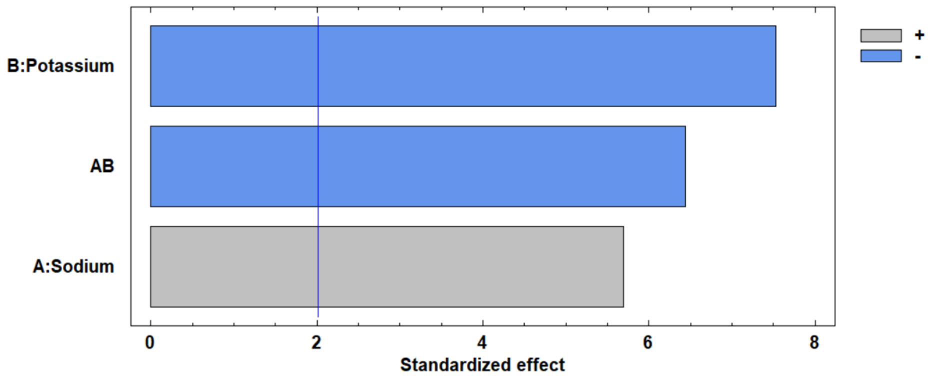

Design of Experiments 2

[illegible]

Model	*RAK_Area*	*RANa_Area*	*Conc_ratio*
Transformation	none	none	none
Model d.f.	3	3	3
P-value	0.0014	0.0265	0.0000
Error d.f.	12	12	12
Stnd. error	0.0219425	0.00568864	0.0000120876
R-squared	71.40	52.32	100.00
Adj. R-squared	64.25	40.40	100.00

				Power at	*Power at*	*Power at*
Coefficient	*Standard Error*	*VIF*	*Ri-Squared*	*SN = 0.5*	*SN = 1.0*	*SN = 2.0*
A	0.335409	1.0	0.0	10.56%	27.89%	78.13%
B	0.33541	1.0	0.0	10.56%	27.89%	78.13%
AB	0.449999	1.0	0.0	8.06%	17.60%	53.32%

alpha = 5.0%, sigma estimated from total error with 12 d.f.

Standardized Pareto Chart for RAK_Area

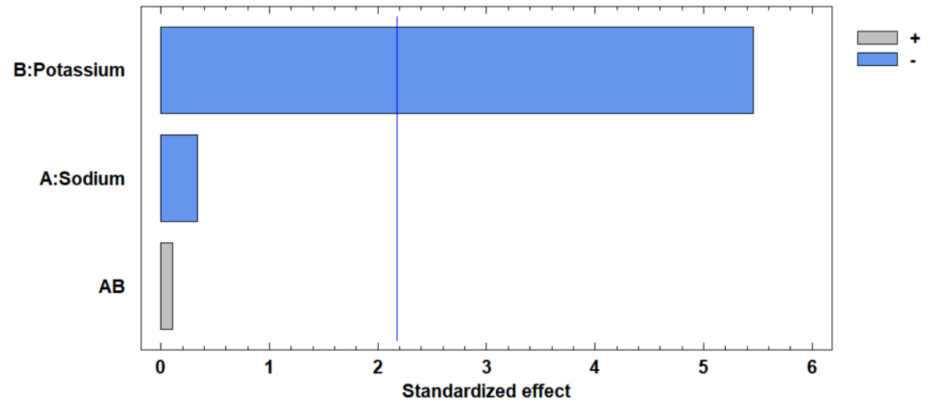

Standardized Pareto Chart for RANa_Area

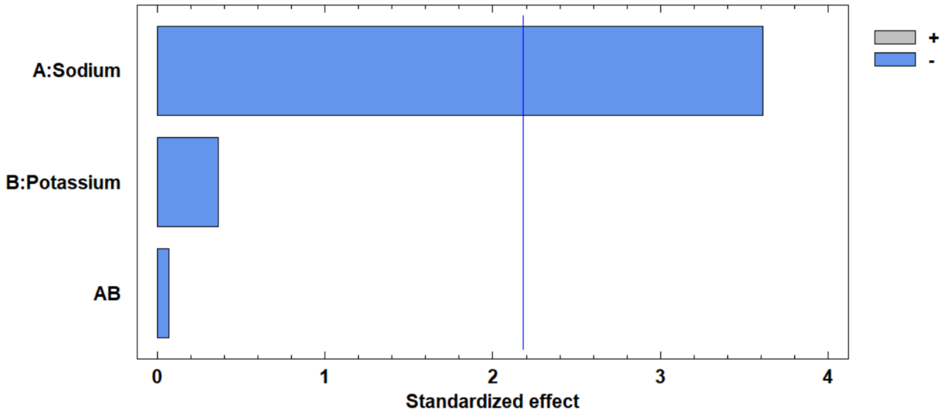

Supplement: Supplementary file 1 [file molecules-29-04882-s001.zip › SUPPLEMENT 1.pdf]
