# Supplementary material for: Virtual Instruments for Peak-Overlapping Studies to Determine Low- and High-Concentration Components with Ion Chromatography: Potassium and Sodium
Source: Molecules. 2024 Oct 15;29(20):4882. doi: 10.3390/molecules29204882 (PMC11510477; doi:10.3390/molecules29204882)

Table S2: Enlarged essential chromatograms of the virtual instruments front panels, decimal sign is comma.

Virtual instrument 1

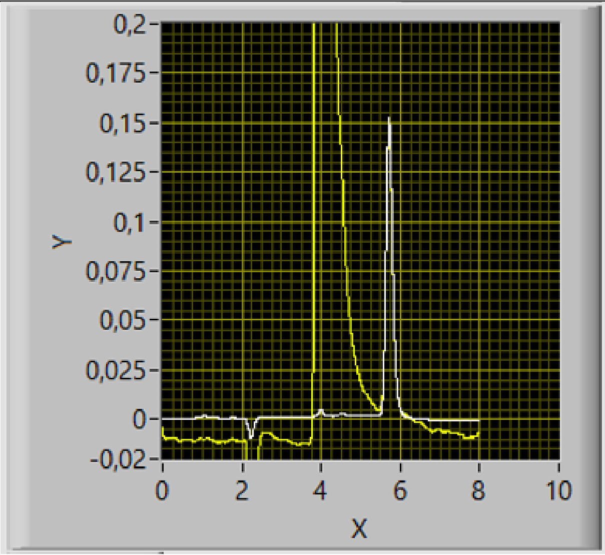

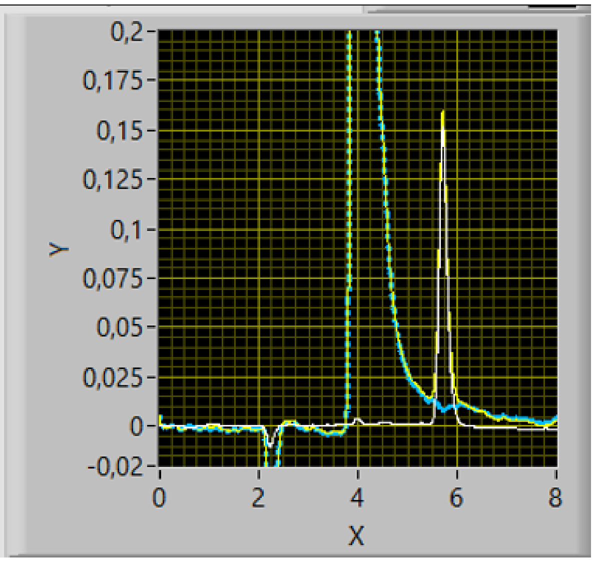

Virtual instrument 2

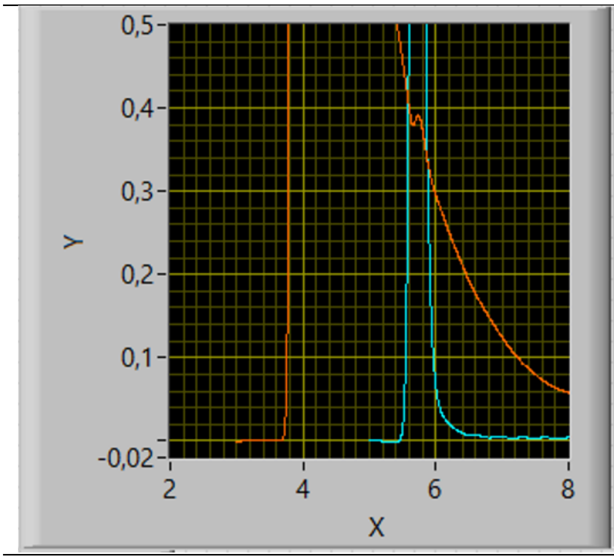

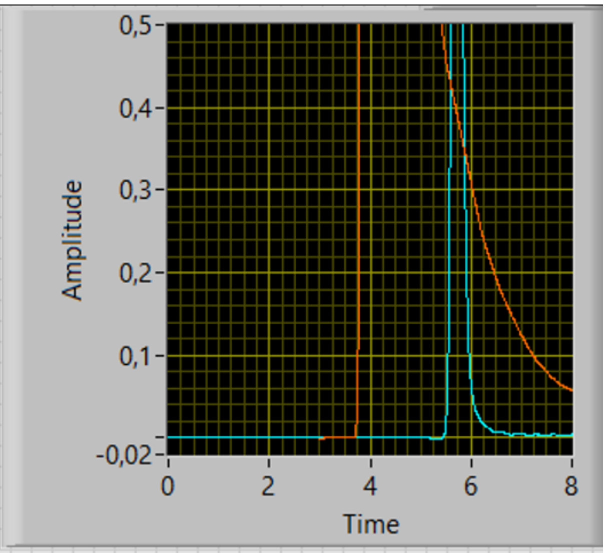

Virtual instrument 3

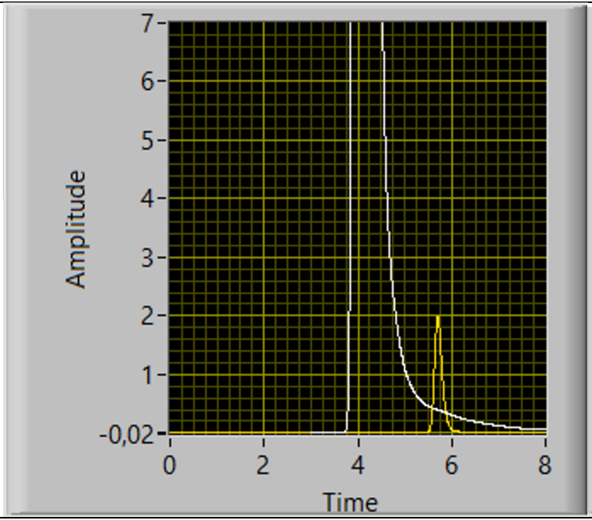

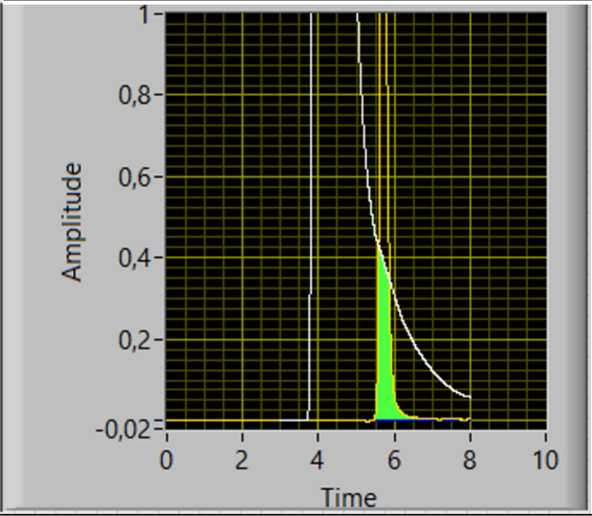

Supplement: Supplementary file 1 [file molecules-29-04882-s001.zip › SUPPLEMENT 2.pdf]
